# Mechanisms of Immune Activation by *c9orf72-*Expansions in Amyotrophic Lateral Sclerosis and Frontotemporal Dementia

**DOI:** 10.3389/fnins.2019.01298

**Published:** 2019-12-10

**Authors:** Kyle J. Trageser, Chad Smith, Francis J. Herman, Kenjiro Ono, Giulio Maria Pasinetti

**Affiliations:** ^1^Department of Neurology, Icahn School of Medicine at Mount Sinai, New York, NY, United States; ^2^Division of Neurology, Department of Medicine, Showa University School of Medicine, Tokyo, Japan; ^3^Geriatrics Research, Education and Clinical Center, JJ Peters VA Medical Center, Bronx, NY, United States

**Keywords:** amyotrophic lateral sclerosis (ALS), frontotemporal dementia (FTD), C9orf72, innate immunity, TDP-43, reactive oxygen species (ROS), therapeutics, microglia

## Abstract

Amyotrophic lateral sclerosis (ALS) and frontotemporal dementia (FTD) are neurodegenerative disorders with overlapping pathomechanisms, neurobehavioral features, and genetic etiologies. Individuals diagnosed with either disorder exhibit symptoms within a clinical spectrum. Symptoms of ALS involve neuromusculature deficits, reflecting upper and lower motor neurodegeneration, while the primary clinical features of FTD are behavioral and cognitive impairments, reflecting frontotemporal lobar degeneration. An intronic G_4_C_2_ hexanucleotide repeat expansion (HRE) within the promoter region of chromosome 9 open reading frame 72 (C9orf72) is the predominant monogenic cause of both ALS and FTD. While the heightened risk to develop ALS/FTD in response to C9orf72 expansions is well-established, studies continue to define the precise mechanisms by which this mutation elicits neurodegeneration. Studies show that G_4_C_2_ expansions undergo repeat-associated non-ATG dependent (RAN) translation, producing dipeptide repeat proteins (DRPs) with varying toxicities. Accumulation of DRPs in neurons, in particular arginine containing DRPs, have neurotoxic effects by potently impairing nucleocytoplasmic transport, nucleotide metabolism, lysosomal processes, and cellular metabolic pathways. How these pathophysiological effects of C9orf72 expansions engage and elicit immune activity with additional neurobiological consequences is an important line of future investigations. Immunoreactive microglia and elevated levels of peripheral inflammatory cytokines noted in individuals with C9orf72 ALS/FTD provide evidence that persistent immune activation has a causative role in the progression of each disorder. This review highlights the current understanding of the cellular, proteomic and genetic substrates through which G_4_C_2_ HREs may elicit detrimental immune activity, facilitating region-specific neurodegeneration in C9orf72 mediated ALS/FTD. We in particular emphasize interactions between intracellular pathways induced by C9orf72 expansions and innate immune inflammasome complexes, intracellular receptors responsible for eliciting inflammation in response to cellular stress. A further understanding of the intricate, reciprocal relationship between the cellular and molecular pathologies resulting from C9orf72 HREs and immune activation may yield novel therapeutics for ALS/FTD, which currently have limited treatment strategies.

## Introduction

Amyotrophic lateral sclerosis and FTD are neurodegenerative diseases with many shared pathologies and symptoms, leading to the belief that they are heterogeneous manifestations along a spectrum. ALS is defined as a motor neuron disease involving corticomotor neuron and corticospinal neuron loss, manifesting in musculature deficits and leading to paralysis and death within 3 to 5 years of diagnosis, often as a result of respiratory failure (Pasinelli and Brown, 2006). Meanwhile, FTD is primarily characterized by degeneration of frontal and temporal lobar regions, leading to impairments in response inhibition, personality, and attention shifting. Clinical overlap exists between the two disorders as the frequency of FTD symptoms can be detected in up to 50% of ALS patients (Lomen-Hoerth et al., 2002; Strong et al., 2003). The prevalence of ALS and FTD symptomatic overlap is far greater in patients with repeat expansions in the G_4_C_2_ promoter of C9orf72 than in patients with sporadic forms ALS; the rate of disease progression in C9orf72 positive patients is also more rapid (Prado et al., 2015). Such overlap in clinical symptoms suggest C9orf72 mutations recruit related pathophysiological pathways responsible for overlapping neuropathological manifestations in ALS and FTD.

Approximately 10% of ALS cases are familial or hereditary. Of the various genetic causes of fALS, repeat expansions of the G_4_C_2_ promoter of C9orf72 account for ∼40% of cases, while the same mutation accounts for 18% of familial FTD cases (Renton et al., 2014; Takada, 2015). The *c9orf72* gene serves active physiological functions in a cell-specific manner. Wild-type *c9orf72* is translated into a guanine nucleotide exchange factor, involved in regulating vesicular trafficking and autophagy in neurons and immune cells (Iyer et al., 2018). In neurons, the proteins generated by *c9orf72* play a passive role in cellular functioning as the selective knockout of *c9orf72* from nestin expressing glia and neurons did not result in motor neuron degeneration, decreased survival, or other pathological hallmarks of ALS suggesting gain of function effects drive c9orf72 toxicity in neurons (Koppers et al., 2015). There is no precise quantity of G_4_C_2_ repeats that can be attributed to a definite diagnosis of ALS. Individuals with c9ALS/FTD have non-coding G4C2 repeats ranging from 66 to over 4400 units. Individuals without ALS typically carry between 2 and 30 repeats in the C9orf72 expansion, suggesting pathology results from excessive repeats (Gijselinck et al., 2016; Balendra and Isaacs, 2018). It is important to emphasize somatic heterogeneity of C9orf72 G_4_C_2_ repeats; the number of repeats quantified in circulating blood cells does not necessarily reflect the number of repeats in microglia or neurons. Pathogenic effects are clearly evident for larger expansions, however, as a linear relationship has been found between the length of the expansion and the rate of disease progression (Byrne et al., 2014). Large repeat pathologic expansions lead to cell-specific deleterious effects on the homeostatic function, including impaired nucleocytoplasmic transport, aberrant RAN translation, production of toxic dipeptide aggregates, and increased oxidative stress. A number of intrinsic and extrinsic cellular mechanisms responsible for recognizing such impairments in cellular activity involve components of the innate immune system.

Toll-like and nod-like receptors are innate immune sensors equipped to recognize moieties of pathogenic materials or imbalances in cellular molecular concentrations or electrical potential, such as those observed in cells from C9orf positive ALS model systems and patients. In particular, the intracellular NOD-, LRR- and pyrin domain-containing protein 3 (NLRP3) inflammasome is unique among pattern recognition inflammasome complexes by its ability to recognize both chemical or electrical disequilibria and toxic protein aggregates, resulting in response the release of pro-inflammatory cytokines including IL-1β and IL-18 from a number of innate immunity cells (Herman and Pasinetti, 2018). A number of studies have described a pathogenic effect of persistent innate immune activation –notably microglia and leukocyte dysfunction– in the development and progression of ALS (Beers and Appel, 2019), and recently studies show activation of innate immune inflammasome complexes may play a contributing role in the pathogenesis in other genetic forms of ALS (McCombe and Henderson, 2011; Lall and Baloh, 2017). As these studies in C9orf72 positive ALS subjects also show that the extent of innate immune activation predicts development and progression of symptoms, it is imperative to define biological and cellular substrates through which C9orf72 expansions promote immune activation.

Large C9orf72 HREs have a pleiotropic effect on normal cellular function; our review will discuss the various molecular pathologies associated with C9orf72 HREs and the subsequent interplay of these effects with the immune system. We further propose that recent evidence showing the role of oxidative stress-mediated innate immune signaling in the pathogenesis of the disease may provide a novel target for therapeutic interventions.

## Neuronal Effects of the C9orf72 Expansion

### Neuropathological Features

Individuals with C9orf72 positive ALS and FTD exhibit distinct region-specific neuropathological features and brain atrophy as assessed by post-mortem analysis. In individuals with ALS who display motor function impairments, C9orf72 repeat expansions preferentially affect the motor neurons of the ventral horn of the spinal cord and pyramidal cells of the corticospinal tract. Neuropathological assessments also find C9orf72 positive individuals with ALS stain positively for TDP-43 exclusively in motor regions (Murray et al., 2011). Meanwhile, cases presenting more on the FTD end of the spectrum show greater pathology and atrophy of neurons located in the frontal and temporal lobes. C9orf72 positive FTD patients can have ALS-like pathology in motor neurons with TDP-43 inclusions, but exhibit more extensive extra-motor pathology (Lee and Huang, 2017; McCauley and Baloh, 2019). Another study found that in six C9orf72 positive cases of FTD, moderate compact neuronal cytoplasmic inclusions were present in the granule cell layer of the hippocampal dentate gyrus, as well as in cerebellar granule cells (Mahoney et al., 2012).

### Nucleotide Secondary and Tertiary Effects of the C9orf72 Expansion

**FIGURE 1:**
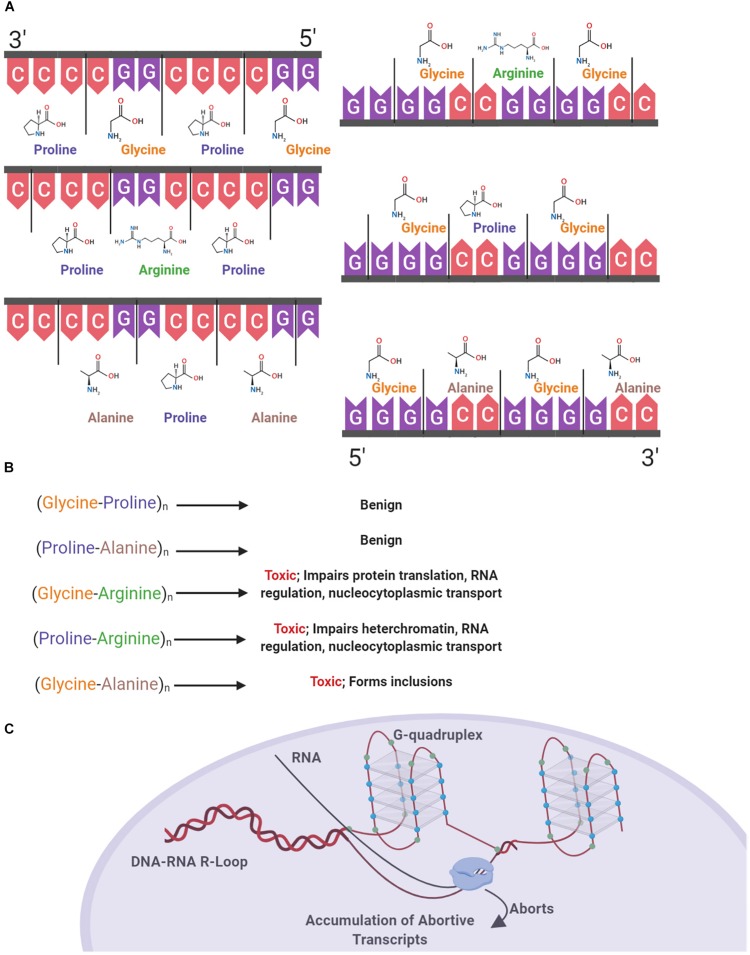
Structural and proteinopathic effects of the C9orf72 hexanucleotide repeat expansion. The C9orf72 HRE consists of an expanded intronic sequence of GGGGCC in the open reading frame 72 of chromosome 9 and produces DRPs in one of three reading frames. DPRs are produced both in the sense (Glycine-Alanine, Glycine-Arginine, Glycine-Proline) and antisense direction (Glycine-Proline, Proline-Arginine, Proline-Alanine) **(A)**. These DPRs have varying degrees of toxicity within the cell, with the arginine containing DPR the most pathogenic **(B)**. The HRE forms a G-quadruplex structure due to hydrogen bonding between guanine bases (blue), and DNA–RNA R Loops form. RNA polymerase is unable to continuously transcribe mRNA, causing the accumulation of abortive transcripts **(C)**.

Secondary and tertiary structural polymorphisms have been shown to occur in both RNA and DNA containing C9orf72 HREs (Kumar et al., 2016). In addition to regulation via proteins, RNA regulation is also mediated by intrinsic mechanisms of translation based upon RNA secondary structures. Guanine-rich intronic regions form highly stable four stranded quadruplex helices that can exist in equilibrium with hairpin structures due to non-covalent hydrogen bond interactions between guanine bases (Zhou et al., 2018). The multiple guanines present in the HRE results in the formation of structural polymorphisms based on G-quadruplexes and hairpins, which previous studies show results in the accumulation of aborted transcripts (Liu et al., 2019). In individuals with C9orf72 expansions, it has been shown that decreased levels of C9orf72 mRNA are present with an abundance of abortive transcripts (van Blitterswijk et al., 2015). Putatively, one may conclude that these structural polymorphisms contribute to the presence of abortive transcripts (Figure 1C).

Structural polymorphisms due to the presence of HREs occurring in DNA predisposes the cell to various pathologic effects, including increased transcription of the antisense strand (Bochman et al., 2012; Fratta et al., 2012). In a study with human cells transfected with G_4_C_2_ repeats, it was shown that DNA replication was impaired due to the presence of G-quadruplexes, with impairment being positively correlated with repeat length (Thys and Wang, 2015). G-quadruplexes also form in the RNA transcribed from the HRE. Consequently, RNA–DNA R-loops form and terminate transcription, causing an accumulation of aborted transcripts which pathologically bind ribonucleoproteins (RNP). RNA G-quadruplexes also bind RNA binding proteins forming an RNA granule or RNA foci (Fay et al., 2017). One of the major ribonucleoproteins bound, nucleolin, has been found to be bound and aberrantly localized due to binding with RNA–DNA R-loops in individuals with C9orf72 mediated ALS, negatively affecting the nucleolus of the cell by causing an aberrant distribution of nucleolin and ultimately nucleolar stress due to the processing of ribosomal RNA and accumulation of untranslated mRNA (Haeusler et al., 2014).

### Transcriptional Consequences of the C9orf72 Expansion

Expanded regions of the HRE in intron 1 of C9orf72 undergo non-canonical repeat-associated non-ATG dependent (RAN) translation both in sense and antisense directions (Cleary et al., 2018). As the HRE sequence undergoes RAN translation, the resulting DPRs accumulate in the cell. With increasing repeat size, efficiency of RAN translation of DPR products also increases (Mori et al., 2013). Proteins are produced as a result of RAN translation in both the sense and antisense direction, with poly-(Glycine-Alanine) (poly-GA), poly-(Glycine-Arginine) (poly-GR), and poly-(Glycine-Proline) (poly-GP) in the sense direction and poly-(Glycine-Proline) (poly-GP), poly-(Proline-Arginine) (poly-PR), and poly-(Proline-Alanine) (poly-PA) in the antisense direction (Figure 1A) (Mori et al., 2013). These accumulating DPR form cytoplasmic inclusions and are located in common sites of neurodegeneration in ALS including the hippocampus, cerebellum, frontal cortex, and motor cortex (Freibaum and Taylor, 2017; Balendra and Isaacs, 2018). Cellular inclusions found in these areas increase in both quantity and size over time, correlating with disease progression (Chew et al., 2019).

The different DPRs have varying levels of toxicity within the cell and contribute to various pathologies (Figure 1B). Poly-GA has been found in the majority of cellular inclusions, putatively owing to the hydrophobic nature of the dipeptide (Kwon et al., 2014). In a study of human post-mortem cerebellar tissues in individuals with the C9orf72 HRE and non-expansion carrying individuals, strong poly-GA signals were only found in individuals with the HRE (Mori et al., 2013). Contributing to the theory of the pathogenicity of poly-GA, an *in vivo* study showed that poly-GA was necessary for the formation of inclusions, by comparing murine models of the full repeat expansion and a repeating vector of Glycine-Arginine DPRs. Mice producing only poly-GR produced a diffuse distribution of DPR deposition, whereas the full HRE produced hallmark aggregates of poly-GR and poly-GA, representative of those found in cases of C9orf72 ALS (Zhang et al., 2018). These poly-GA containing inclusions are predominantly distributed throughout the cytoplasm, with a limited amount forming nuclear inclusions. Presence of inclusions leads to an increase in stress in the endoplasmic reticulum, impairment of the ubiquitin-proteasome system, and ultimately leading to an increase in caspase-3, an indicator of cell death (Zhang Y.J. et al., 2014).

Arginine containing DPRs are the most pathological to the cell (Xu and Xu, 2018). *In vivo* models of fruit flies expressing a single variant of DPR demonstrate the relative toxicity of both poly-GR and Poly-PR when compared to alanine containing species. Fruit flies with expressing either 100 repeats of GR or 100 repeats of PR both had significantly decreased survival compared to alanine containing variants (Mizielinska et al., 2014). Poly-GR has been shown to have toxic effects on ribosomal proteins, by co-localizing with various ribosomal proteins and binding translation initiation factors, including eIF3η, in murine models as well as post-mortem C9orf72 ALS brain tissue (Lee et al., 2016; Zhang et al., 2018). Models solely comprised of repeating GR units were not found to form cellular inclusions perhaps due to the hydrophilic nature of the dipeptide (Kwon et al., 2014; Zhang et al., 2018). Like poly-GR, poly-PR does not aggregate due to being hydrophilic. However, the arginine residue may indicate that the protein is readily shuttled back to the nucleus due to the common post-translational methylation via arginine methyltransferases (Herrmann and Fackelmayer, 2009). Poly-PR has been shown to have deleterious effects on heterochromatin formation within the cell. *In vivo* models expressing only the proline-arginine dipeptide show severe mortality compared to control, with approximately 60% of mice dying by 4 weeks of age. Mechanisms of toxicity attributed to this dipeptide include gene silencing via histone modifications and the accrual of double stranded RNA (Zhang et al., 2019). Furthering the notion of the toxicity of the arginine containing DRPs, in human neurons cultured and transfected with different DPRs, it was shown that both poly-GR and poly-PR significantly impair translation (Moens et al., 2019).

### Nucleocytoplasmic Transport Impairments in Response to C9orf72 Expansions

The mislocalization of nuclear proteins to the cytoplasm is one of the common findings in C9orf72 mediated ALS. Common proteins found to be aberrantly localized include RNA binding proteins RBPs serve as an important mediator in the process of post-transcriptional control of RNAs by participating in the transportation and splicing of mRNA, as well as RNA metabolism. RBPs are known to be impaired in C9orf72 ALS include TAR DNA Binding Protein of 43 kDa (TDP-43) and HNRNPA1 (Zhao et al., 2018; Prasad et al., 2019).

TDP-43 containing inclusions are found in approximately 90% of ALS cases, regardless of genetic or sporadic etiology (Mitchell et al., 2015). The basal function of the protein serves as a transcription regulator and a factor in post-transcriptional modifications such as alternative splicing (Winton et al., 2008). While location of TDP-43 typically constantly shifts between the nucleus and cytoplasm, it has been noted that in cases of ALS there is an increase in the cytoplasmic concentration of the protein due to impaired nucleocytoplasmic transport (Winton et al., 2008). In a study utilizing brain tissue from hexanucleotide repeat carrier individuals, it was shown that there was a strong correlation between the presence of TDP-43 inclusions and degree of neurodegeneration. DPR formation and accumulation are thought to precede the appearance of TDP-43 inclusions, as evidenced by the discovery of DRPs sometimes being found as a central component of TDP-43 inclusions (Mackenzie et al., 2013). *In vivo* Drosophila studies have interrogated the relationship between pathologic DPRs and TDP-43. In a fly model producing only HRE RNA and which does not undergo RAN translation, TAR DNA-binding protein-43 homolog (TBPH), the fly equivalent of TDP-43, was not found to be aberrantly mislocalized to the cytoplasm. However in fly models producing DPR, TBPH was found to be localized to the cytoplasm (Solomon et al., 2018). In a human study utilizing brain tissue from hexanucleotide repeat carrier individuals, it was shown that there was a strong correlation between the presence of TDP-43 inclusions and degree of neurodegeneration. DPR formation and accumulation are thought to precede the appearance of TDP-43 inclusions, as evidenced by the discovery of DRPs sometimes being found as a central component of TDP-43 inclusions (Mackenzie et al., 2013).

## Effects of C9orf72 Expansions on Immune Cells

In contrast to the gain of function effects of C9orf expansions in neurons, C9orf72 expansions in innate immune cells result in loss-of-function toxicity via impairment of cellular homeostatic processes including autophagy (Lall and Baloh, 2017). C9orf72 expression is particularly high in the dendritic immune cells and microglia, the resident innate immune cells of the brain, suggesting consequences of C9orf72 expansions differ based on cellular phenotypes (Zhang Y. et al., 2014; O’Rourke et al., 2016). C9orf72 mutations may have consequential effects on the regulation of synapses by microglia and may cause persistent microglial activation that has a pathogenic effect, exacerbating the progression and development of ALS. We will therefore review the evidence to support the claim that biological pathomechanisms induced by C9orf72 expansions alter immune activity to the consequence of neuronal health.

### Activation and Distribution of Microglia

Microglia are resident innate immune cells of the brain. Microglia are derived from the initial primitive hematopoietic process in the extra embryonic yolk sac and migrate during fetal development to reach their final destination in the central nervous system. Evidence indicates their neuronal population is maintained through self-renewal throughout the lifespan (Dubbelaar et al., 2018; Li and Barres, 2018). Under homeostatic conditions microglia exist along an immunophenotypic spectrum between one of two overarching states: a surveillant phagocytic state and an activated pro-inflammatory state. While in a surveillant state microglia survey the neuronal environment, acting as resident brain sentinel cells, and monitor and prune synaptic connections (Shemer et al., 2015). Upon recognition of sterile or pathogenic stress signals microglia adopt a proinflammatory profile defined by changes to their transcriptional profiles, by the upregulation of functional immune genes such as major histocompatility class complexes, Iba1 and CD86, and by the production and secretion of cytokines and free radicals that affect neuronal function (Lall and Baloh, 2017; Mammana et al., 2018; McCauley and Baloh, 2019). While microglia activation may serve a beneficial immediate role in clearing synaptic debris and pathogens, persistent microglia activation and inflammation have detrimental collateral effects on neuronal function. Numerous studies describe how microglia recognize and maintain an inflammatory immunophenotype in response in extracellular protein inclusions noted in other neurodegenerative disorders, including β-amyloid in Alzheimer’s disease, and α-synuclein in Parkinson’s disease (Kreutzberg, 1996).

Common among various etiologies of ALS, there is evidence that microglia adopt an inflammatory morphological state that predicts disease progression. Histological studies using post-mortem brain samples from ALS patients find that resident microglia increase in their population in proportion to the stage of disease progression (Geloso et al., 2017). Moreover, post-mortem brain samples from individuals with C9orf72 positive ALS find a positive correlation between the magnitude of expression of CD86 and Iba1, markers of microglia activation and proliferation, and the severity of ALS symptomology and magnitude of TDP-43 deposition (Brettschneider et al., 2012). Notably, this study showed that microglial pathology in the motor cortex was more severe in C9orf72 positive ALS than in cases of sporadic ALS. Additional *post-mortem* brain analysis of multiple white matter regions including the motor cortex confirm that microglia immunoreactivity is greater in individuals with C9orf72 mediated ALS compared to cases of sporadic ALS based on Iba1 and CD68 staining (Rostalski et al., 2019). Persistent region specific patterns of microglial activation in ALS is also demonstrated by the utilization of positron emission tomography (PET) scans in individuals with ALS. An injection of a radioactive tracer that labels the translocator (TSPO) protein in functionally immunoreactive microglia results in significantly higher signal intensity in primary motor, supplementary motor, and temporal areas of the brain (Corcia et al., 2012). These investigations indicate an immunoreactive microglia inflammatory phenotype that can have cytotoxic consequences is a component of ALS pathogenesis, and in particular for the C9orf72 phenotype. Whether activation of microglia is consequence of neurodegeneration or an instigator of it remains under investigation.

C9orf72 expression is higher in microglia than in any other cell type, including neurons and research suggests the wild type *c9orf72* gene plays a central role in maintaining immune homeostasis (O’Rourke et al., 2016). Meanwhile, in myeloid lineage cells, the wild type *c9orf72* gene serves an active role in maintaining immune homeostasis. In mice deficient for the *c9orf72* gene, it has been shown that there is upregulation of genes linked to inflammatory responses. Moreover, microglia isolated from these animals show increased levels of pro-inflammatory cytokines IL-6 and IL-1β. Further indicative of a pro-inflammatory state, hyperplasia of both the spleen and lymph nodes were observed in *c9orf72* knockout animals. Animals with this knockout did not directly display neurodegeneration, however it has been hypothesized that a lack of functional C9orf72 in microglia can cause defects in their ability to remove aberrantly folded proteins (O’Rourke et al., 2016).

Microglia isolated from a number of additional murine ALS model systems exhibit higher inflammatory potential as well. Microglia cultured from late stage mutant SOD1 mice were shown to have decreased mRNA levels of proteins associated with the anti-inflammatory end of the activation spectrum as well as increased levels of RNA for genes involved in the generation of ROS when compared to microglia cultured from the same mice early on in the progression of the disease. End disease stage microglia expressed an increase in levels of NOX2 mRNA, a component of NADPH oxidase responsible for the generation of superoxide, compared to microglia from mice in the initial stages of ALS. To further interrogate the contribution of microglia in neurodegeneration, microglia from both early and late stage ALS were co-cultured with motor neurons, and compared to motorneurons co-cultured with wild-type microglia. Motorneurons co-cultured with microglia from end stage animals exhibited increased cell death, and decreased neurite count when compared to both beginning stage microglia and wild type microglia co-cultures, indicative of the toxic role microglia may take on as the disease progresses (Liao et al., 2012; Geloso et al., 2017). Further *in vivo* studies of SOD1 mice have shown that there is upregulation of inflammatory genes such as *Apoe and Csf1* early on in the disease state, suggesting the role of neuroinflammation in the pathogenesis of ALS (Butovsky et al., 2015). Spinal cords from mutant mice additionally show microglial activation before symptoms of myasthenia are present which proceeds through the development of symptomology, indicating a temporal correlation (Volonté et al., 2019). Moreover, a pathological role for microglia dysfunction in ALS/FTD is further suggested in FTD by progranulin mutations and from variants in *TREM2*, a microglia expressed gene that increase susceptibility for ALS (Cruts et al., 2006).

### Glial Reactivity

Excessive glial reactivity has been theorized to play a contributing role in the pathogenesis of ALS. In an *in vitro* study involving the culturing of primary motor neurons and microglia, when microglia were activated by human ALS immunoglobulin G (IgG), microglia transitioned to an activated state and damage to motor neurons occurred, leading to significant neuronal loss. Surviving motor neurons were found to have a smaller sized soma, fewer neurites, and a decrease in arborization (Zhao et al., 2004). Reinforcing this notion, the same results were replicated when microglia were incubated with LPS rather than ALS IgG, indicating an increased and pathological response originating from microglia. This same damage did not occur when a culture of only motor neurons was exposed to either LPS or ALS IgG, suggesting that the presence and activity of microglia is necessary for neurodegeneration to occur. Furthermore when incubated with an inhibitor of nitric oxide was added to culture prior to addition of either LPS or ALS IgG; motor neuron survival was greatly increased, strengthening the evidence supporting the toxicity of ROS in the pathology of ALS (Zhao et al., 2004). To investigate the role of basal state microglia in SOD1 familial ALS, PU.1^–/–^ mice, devoid of macrophages, neutrophils, T cells, B cells, and microglia were cross bred with SOD1^*G93A*^ mice. When transplanted with basal state wild-type microglia, disease progression was slowed and survival increased when compared to both SOD1^*G93A*^ mice with functioning, SOD1^*G93A*^ microglia, and SOD1^*G93A*^/PU.1^–/–^ mice, without microglia. *In vitro* studies were further carried out, comparing the effects of SOD1^*G93A*^ microglia on motor neurons. Wild type microglia produced less ROS, RNS, and neuronal death occurred when compared to SOD1^*G93A*^ mutation carrying microglia (Beers et al., 2006). In totality, these experiments further reinforce the notion of aberrant microglia cells contribution to the development of ALS pathology.

While resident microglia reside within the central nervous system, additional macrophages and monocytes reside outside of the CNS and are able to infiltrate and respond to disturbances (Mammana et al., 2018). Additional tissue resident cells of myeloid lineage include perivascular macrophages, meningeal macrophages, and macrophages of the choroid plexus. In addition to the increase in population of tissue resident microglia, infiltration of monocytes is also apparent, with cell populations identified by the expression of C-C chemokine receptor 2 (CCR2) in monocytes which is absent in microglia (Mammana et al., 2018). Chemokines produced by microglia, among other cells, include the C-C Motif Chemokine Ligand 2 (CCL2), which is produced during neuroinflammation and may serve as the cell population responsible for the attraction of monocytes expressing CCR2 (Shemer et al., 2015). Previous studies show that in disease states when the blood–brain barrier is compromised, monocytes with transcriptional profiles distinct from the tissue resident microglia penetrate the CNS and participate in response to damage (Li and Barres, 2018; McCauley and Baloh, 2019). Damage to the blood brain barrier has been previously demonstrated *in vivo* with SOD1 mice, as well as in *post-mortem* examination of sALS brain tissue (Garbuzova-Davis and Sanberg, 2014).

Astrocytes, the other dominant cell population in the brain, also exhibit susceptibility to C9orf72 expansions. Astrocytes are responsible for providing metabolic support to neurons, axon maintenance, protection against oxidative stress, and the regulation of neuroendothelial permeability (Bélanger et al., 2011; Garwood et al., 2017). In a murine model of a 149-repeat G_4_C_2_ expansion, it was shown that elevated levels of GFAP, a marker for astrocytes, preceded the cortical thinning as evidenced by NeuN + staining; the increase in GFAP immunoreactivity preceded the onset of cortical thinning at 6 months by a number of months (Chew et al., 2019). A number of putative mechanisms that link astrogliosis with the onset of ALS disease pathology have been investigated. *In vitro* evidence from human induced astrocytes from C9orf72 patients suggests that dysregulation of astrocyte miRNA involved in the regulation of axonal maintenance genes impairs extracellular trafficking between astrocytes and neurons, leading to motor neuron death (Varcianna et al., 2019). Other recent studies find that induced astrocytes from C9orf72 positive fALS and sporadic ALS individuals exhibit loss of metabolic flexibility, in particular in glucose and fructose metabolism (Allen et al., 2019). A major point of future interrogation lies in understanding how impaired astrocyte function activity may particularly increase susceptibility for motor neuron degeneration.

## Interplay of c9orf72 Expansion Pathophysiologies and Immune Activation

As described above c9orf72 HRE elicits a number of pathophysiological consequences, impairing both neuronal and immune cell function. We illustrate the gain-of-function effects of C9orf72 HREs in neurons and loss-of-function effects of C9orf72 HREs in immune cells. Presented below is evidence that effects of c9orf72 HREs in neurons have a complex, reciprocal, relationship with its effects in the immune system, with pathophysiologies generated in one cell type influencing and exacerbating effects of HREs in others. Dysfunction due to C9orf72 pathology may therefore create a self-perpetuating cycle in which expansion related effects trigger chronic immune action, causing further cellular dysfunction.

### Reactive Oxygen Species

Other forms of familial ALS have directly implicated the pathologic nature of excessive ROS production or impaired breakdown. SOD1 normally functions to catalyze the reaction of superoxide into oxygen and hydrogen peroxide, which then is able to diffuse through lipid cell membranes and cause direct damage in cells (Ma et al., 2017). SOD1 mutations have been linked to a large number of fALS cases via various pathological mechanisms. Aberrant production and breakdown of ROS have been shown to occur in both sporadic and fALS, with these molecules targeting the NMJ in cases of both sporadic ALS and SOD1 mediated fALS. High levels of ROS have been shown to impair synaptic transmission in the NMJ via the depletion of presynaptic neurotransmitters available for release and upregulation of calcium levels within the terminal. Ultimately, later during disease progression, the nerve terminal shrinks and acetylcholine release is impaired (Pollari et al., 2014). Impairment within the NMJ initially presents as weakness in the muscles and ultimately results in paralysis (Campanari et al., 2016).

In addition to the release of cytokines, microglia produce ROS including hydrogen peroxide and superoxide, as well as RNS including nitric oxide. An overproduction of ROS or dysfunction in the breakdown of ROS leads to a state of oxidative stress in the cell (Kim et al., 2015). Excessive production of superoxide, one of the ROS implicated in the pathogenesis of ALS, can cause oxidative stress either directly or indirectly by creating secondary free radicals (Ma et al., 2017). Excess levels of ROS can catalyze the formation of other molecules into ROS, creating a cycle of ROS generation. These endogenous ROS can go on to act on the various macromolecules within the cell, including lipids and proteins. Reactions that involve hydroxyls include the addition of carbonyls to amino acids, making them susceptible to proteolysis and reactions inducing the change of DNA bases making strands susceptible to breaks (Betteridge, 2000).

Reactive oxygen species biomarkers have additionally been suggested as a quantitative measure of disease progression. Human testing in individual with ALS has revealed NfL, 4-hydroxy-2-non-enal (4-HNE), and 8-oxo-2′-desoxyguanosine (8-oxo-dG), to demonstrate the ability to measure not only disease progression, but differentiate individuals with either a slow or fast disease progression. These markers indicate various downstream effects of oxidative stress including axonal health, DNA oxidation, and lipid peroxidation respectively (Devos et al., 2019). Post-mortem specimens from individuals with SOD1 linked fALS and individual with spontaneous ALS, elevated oxidative damage markers OH^8^dG were found to occur in neurons from both sporadic ALS and familial ALS patients, suggesting ROS and microglia pathology as a commonality between forms of sporadic and fALS (Ferrante et al., 1997). Based upon longitudinal studies of serum cytokine levels in individuals with ALS, it appears that IL-6, TNF-α, and IFN-γ show the strongest correlation with ALS pathology (Lu et al., 2016). These heightened levels of inflammatory cytokines have various toxic effects on the cell. High levels of TNF-α have been shown to induce the formation of ROS via the activation of NADPH oxidase (Fischer and Maier, 2015). The resulting high levels of NADPH oxidase has been linked to neurodegeneration, putatively indicating a potential target for novel therapeutics (Gao et al., 2012).

### Oxidative Stress and RAN Translation

**FIGURE 2:**
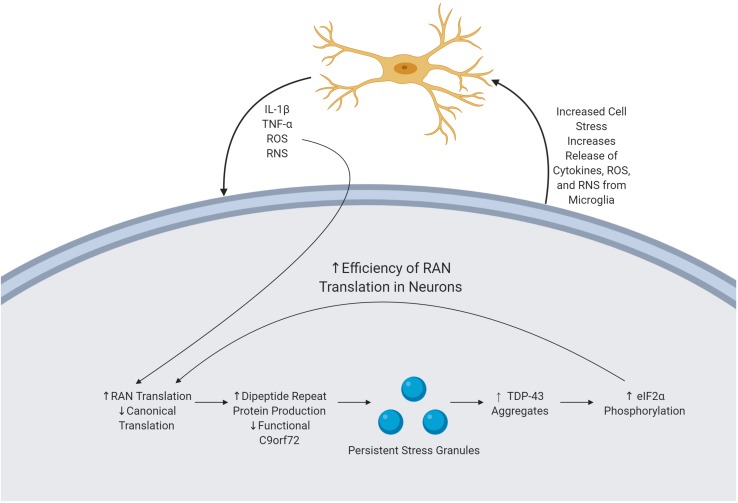
Mechanism of immune activation by byproducts of the C9orf72 repeat expansion. C9orf72 pathology is driven by the presence of an expanded HRE, producing various cellular pathologies. DRPs are produced via RAN translation and subsequently accumulate, causing cellular stress. In response, the cell forms SGs which are unable to readily dissolve. TDP-43 is subsequently recruited to these SGs, and there is upregulation of eIF2α phosphorylation thereby increasing the efficiency of RAN translation, thereby perpetuating the cycle. Microglia sense neuronal stress and in response release various cytokines and ROS. ROS increase the efficiency of RAN translation, furthering the feedback loop of pathology and neurodegeneration.

During periods of increased stress, cells often rely upon atypical forms of translation. The integrated stress response, a process by which the cell responds to stressors including oxidative stress, has been implicated in cases of C9orf72 mediated ALS. When undergoing the integrated stress response, cells commonly reduce canonical translation. In response to the presence of oxidative stress, cells with the C9orf72 HRE increase levels of non-canonical RAN translation of DRPs (Figure 2) (Westergard et al., 2019). In order to do so, initiation factors including eIF2α are phosphorylated, which has the effect of reducing the initiation of canonical translation, ultimately increasing RAN translation, although the mechanism by which RAN translation efficiency is altered has not yet been determined (Cheng et al., 2018). This increased translation of DPRs can lead to an increase in pathologies, ultimately accelerating neurodegeneration.

### Persistent Stress Granules

Stress granules (SGs) are essential in mediating the response of the cell to environmental stress and subsequently stopping the buildup of aberrant, misfolded proteins (Chen and Liu, 2017). SGs are comprised of active mRNA and RBPs; the formation of the SG is believed to serve as a protective measure by the cell to prevent damage to these structures (Colombrita et al., 2009). Following the removal of the triggering stimuli, some SGs independently dissolve their structure, while others must undergo autophagy (Kedersha and Anderson, 2007; Chitiprolu et al., 2018). A major initiator in the formation of SGs is the presence of oxidative stress; SGs form within minutes of exposure to ROS (Kim et al., 2015; Chitiprolu et al., 2018). The presence of non-dissolving SGs in the cell has been linked to a persistent state of cellular stress. TDP-43 inclusions, pathognomonic for C9orf72 mediated ALS, are thought to exert a pathological effect through recruitment to SGs. TDP-43 pathology has been linked to the upregulation of eIF2α phosphorylation, thereby increasing the efficiency of RAN translation and the subsequent production of DPRs (Figure 2) (Kim et al., 2014).

*In vitro* experiments investigating the formation of SGs in response to oxidative stress have demonstrated that C9orf72 was routinely recruited into SGs. Low concentrations of the normal functioning C9orf72 gene product are commonly found in C9orf72 ALS. Reasons for this decrease include the production of abortive transcripts, DPRs, and dysregulated protein synthesis. Due to the low concentration of functional C9orf72, SGs less readily dissipate and there is an increase in the accumulation of TDP-43 aggregates (Sellier et al., 2016; Chitiprolu et al., 2018). Furthermore, decreased levels of normal C9orf72 gene product, as is found in individuals with C9orf72 ALS, increases cellular sensitivity to stressors via the impairment of assembly and dissolution of SGs (Maharjan et al., 2017). The persisting state of SGs in the cell impairs RNA metabolism and protein degradation, leading to the aggregation of aberrant proteins; a common finding in C9orf72 ALS.

### Inflammasome Activation

As indicated above, the NLRP3 inflammasome is part of a family of intracellular innate immune sensors that are integral for cellular defense. Comprised of NLRP3, ASC, and Caspase-1, activation of the NLRP3 inflammasome complex involves a two-step paradigm: a priming signal is required to generate *nlrp3* transcription, while a second activation signal results in assembly of the oligomeric complex. Ultimately, the activation of the NLRP3 inflammasome leads to the activation of Caspase-1, which in turn activates pro-inflammatory cytokines such as IL-1β and IL-18. In certain cells, activation of inflammasome complexes results in pyroptosis, a form of inflammatory mediated cell death in which proinflammatory cytokines are released and the cell response is amplified and propagated (Swanson et al., 2019). It is critical to note that NLRP3 inflammasome activation can be achieved by a number of pathophysiological pathways generated by C9orf72 HREs previously described. These include lysosomal dysfunction, mitochondrial functional impairments, intracellular metabolic imbalances, and intracellular protein aggregates. TDP-43 inclusions, a pathomechanism noted in patients with genetic forms of TDP-43 ALS, readily activate the NLRP3 inflammasome in primary microglial cultures, resulting in increased production of IL-1β. Interestingly, motor neurons exposed directly to TDP-43 do not exhibit neurotoxicity; the presence of microglia exposed to extracellular TDP-43 protein and subsequent secretion of proinflammatory cytokines is necessary for neurodegeneration to occur (Zhao et al., 2015). Similar increases in expression of NLRP3 inflammasome components are also present in cases of post-mortem tissue from individuals with sporadic ALS, with significant upregulation of ASC and IL18 (Johann et al., 2015). Additionally, elevated serum levels of IL-18 have been demonstrated in cases of sporadic ALS, thereby suggesting the possible upregulation of an upstream component of the inflammasome (Italiani et al., 2014).

Recently, the activation of the NLRP3 inflammasome was directly investigated in SOD1^*G93A*^ mice. Spinal cord from animals with late stage disease showed significant upregulation of components of the NLRP3 inflammasome, including *nlrp3*, *pro-IL-1*β, *ASC*, and caspase-1. In this study, NLRP3 was also shown to be expressed in both microglia and astrocytes. To further determine the ubiquity of NLRP3 activation in models of ALS, the authors also analyzed spinal cord tissue for gene expression in TDP-43 mutant mice and similarly displayed a significant upregulation of NLRP3 inflammasome components NLRP3, Caspase-1, and ASC. In addition to these *in vivo* investigations, primary microglia cultures from both wild type mice were obtained and incubated in soluble SOD1^*G93A*^. IL-1β release was found to occur in a dose-dependent manner in these cells, however when MCC950, a specific NLRP3 inhibitor, was added, cells significantly reduced their secretion of IL-1β. The same paradigm was conducted in wild-type cells incubated with mutant TDP-43 protein. Microglia were found to secrete IL-1β when stimulated with TDP-43 protein, and the effect was again abolished by the addition of MCC950 (Deora et al., 2019). This demonstration of the activation of the NLRP3 inflammasome in multiple *in vivo* and *in vitro* models of ALS, and the subsequent reduction in IL-1β secretion with an NLRP3 inhibitor suggests a commonality that may potentially be utilized as a novel target for therapeutics.

To maintain proper homeostasis, a fine balance must be struck between appropriate and disproportionate inflammatory responses to triggering stimuli. Excessive or chronic activation of the innate immune system has been linked to increased cellular stress and degeneration of motor neurons. This nexus of innate immune activation and neuronal health may serve as a potential target for novel therapeutics.

## Therapeutic Approaches

### First Line Treatments

Currently, only two medications are on the market for the treatment of ALS: riluzole and edaravone (Zhang et al., 2012; Cruz, 2018) (Table 1). Riluzole was the first drug to be approved for the treatment of ALS by the United States Food and Drug Administration and acts via the modulation of glutamatergic transmission. Effects from treatment with riluzole are modest, with median survival time increasing from 2.25 years in non-treated patients to 3.07 years in treated patients (Mitchell et al., 2009). The most recently FDA approved treatment for ALS, edaravone, was previously approved as a treatment for ischemic stroke. In cases of ischemic stroke, large concentrations of inflammatory cytokines and reactive species are released, after which neuroinflammation and ultimately neurodegeneration occur (Yuan et al., 2014). Although the mechanism of action of edaravone has not been fully elucidated, edaravone is believed to act as a ROS scavenger and decrease the generation of ROS (Zhang et al., 2012; Cruz, 2018). In an *in vivo* study of rats with ischemic stroke treated with edaravone, it was shown that activated microglia produce less TNF-α, IL-1β, ROS, and iNOS compared to rats treated with vehicle (Yuan et al., 2014). Clinical trials of edaravone additionally proved to be successful in both slowing reduction in ALS functional rating scale (ALSFRS-R) score, as well as in the decrease in markers of oxidative stress in cerebrospinal fluid over the course of a 6 month course of treatment (Yoshino and Kimura, 2006; Luo et al., 2019).

**TABLE 1:** Overview of accepted therapeutics and trials targeting inflammation.

**Drug**	**Target**	**Outcome**	**References**
AAD-2004	COX inhibitor	Efficacy *in vivo*	Shin et al., 2012
Anakinra	IL-1 receptor antagonist	Efficacy *in vivo*, preclinical trials inconclusive	Maier et al., 2015
Aspirin	Non-selective COX inhibitor	Efficacy in case-control study	Tsai et al., 2015
Celecocoxib	Selective COX-2 inhibitor	Unsuccessful in clinical trials	Cudkowicz et al., 2006
Edaravone	ROS	FDA approval; first line treatment	Zhang et al., 2012; Cruz, 2018
Minocycline	Anti-inflammatory (mechanism unknown)	Efficacy *in vivo*, unsuccessful in phase III	Kriz et al., 2002; Keller et al., 2011
Niclosamide	S100A4 inhibitor	Efficacy *in vitro*	Serrano et al., 2019
Nimesulide	Selective COX-2 inhibitor	Efficacy *in vivo*	Pompl et al., 2003
NP001	NF-κB	Efficacy in subset of patients in phase II trials	Miller et al., 2014, 2015
Riluzole	Glutamatergic transmission	FDA approval; first line treatment	Mitchell et al., 2009

*The nexus of neuroinflammation and the pathogenesis of ALS has been a target for the development of novel therapeutics. Currently, there are two FDA approved treatments for ALS: Riluzole, which targets glutamatergic transmission, and edaravone, which is believed to target ROS (Mitchell et al., 2009; Cruz, 2018). The reciprocal relationship of inflammation and neurodegeneration and a large case-control study showing an inverse relationship between individuals taking Aspirin and the incidence of ALS has yielded various preclinical and clinical trials into both selective and non-selective COX inhibitors (Pompl et al., 2003; Cudkowicz et al., 2006; Shin et al., 2012; Tsai et al., 2015). Furthermore, other components of the inflammasome have been targeted in recent times with some success both in clinical and preclinical trials (Maier et al., 2015; Miller et al., 2015; Serrano et al., 2019).*

### Aspirin, Aspirin Derivatives, and Combating Inflammation

Mitigating neuroinflammatory sequelae as a therapeutic target is a promising strategy in developing novel therapeutics for ALS. Cyclooxygenase (COX) serves an essential role in inflammation pathways and has long been a target for medical treatments. COX exists as two separate isozymes, COX-1 and COX-2 which are responsible for the anabolism of integral molecules in inflammatory pathways such as prostaglandins (Vane and Botting, 2003; Fitzpatrick, 2004). Inhibition of the COX-2 isozyme has been demonstrated as an effective treatment strategy in *in vivo* models of ALS (Drachman et al., 2002; Pompl et al., 2003). The preferential COX-2 inhibitor, nimesulide, was shown to be efficacious in a SOD1 linked model of fALS both in terms of delaying motor impairment and decreasing levels of prostaglandin-E2 (Pompl et al., 2003). Unfortunately, selective COX-2 inhibitors such as celecoxib have so far not demonstrated efficacy over placebo treatment in clinical trials (Cudkowicz et al., 2006). This may however be related in part to the difficulty in establishing a diagnosis prior to the development of symptoms, during the period in which inflammation is present, but neurodegeneration has not yet begun.

One of the most commonly used non-selective COX inhibitors, Aspirin (acetylsalicylic acid), has been shown to have a potential use for various disorders and illnesses ranging from preventing myocardial infarction to treating psychiatric illness (Barbarawi et al., 2019; Müller, 2019). Aspirin salts previously have been investigated as potential therapeutics for ALS in transgenic SOD1 mice. When treatment was started early enough, motor pathologies were delayed, however end stage disease was ultimately not prevented (Barnéoud and Curet, 1999). In addition to the ability of aspirin to inhibit prostaglandin synthesis, it has also been proposed that aspirin can serve a role in the scavenging of ROS, specifically hydroxyl radicals (Aubin et al., 2002). In a total population based case-control study of individuals in Taiwan, aspirin use was found to have an inverse correlation to the incidence of ALS when controlling for confounders such as steroid use (Tsai et al., 2015). Other monophenolic acids, including structural isomers of the key active metabolite of aspirin, salicylic acid, have shown efficacy as anti-inflammatory and neuroprotective agents, with success specifically against hydrogen peroxide mediated damage (Winter et al., 2017).

Other derivatives of aspirin aimed at mitigating neuronal injury via neuroinflammation have shown promise in delaying the progressive neurodegeneration of ALS. One such novel compound, 2-hydroxy-5-[2-(4-trifluromethylphenyl)-ethylaminobenzoic acid] (AAD-2004) has been utilized in SOD1 transgenic mice to combat the inflammatory sequelae of ALS. When administered with AAD-2004, degeneration of motor neurons was decreased, as was production of free radicals, and activation of microglia in the spinal cord. Perhaps most promisingly, AAD-2004 was shown to be more efficacious than the currently approved treatment riluzole at delaying onset of disease symptoms, improving motor function, and increasing survival (Shin et al., 2012).

Additional attempts at targeting inflammatory effects of ALS include the use of minocycline, an antibiotic with anti-inflammatory effects. When administered in a murine model of SOD1*G37R* ALS, motor neuron degeneration was delayed and survival was increased. Additionally, less microglial activation in spinal cord motor neurons was observed both during initial states of the disease as well as in the final stages (Kriz et al., 2002). Although minocycline ultimately proved ineffective in stage III clinical trials, findings did suggest that the microgliosis persisting in the latter stages of the disease may be resistant to treatment: thereby underlying the importance of preventing the initial stages of microgliosis. To test this hypothesis, minocycline administration was initiated at varying time points of ALS disease state in a SOD1 mouse line. While treatment initiated after the onset of pathology did not have a positive effect on survival or motor symptoms, animals pretreated with minocycline were shown to have a greater mean survival time, highlighting the necessity of preventing the initial stages of inflammation before neurodegeneration can begin (Keller et al., 2011).

Targeting IL-1β, an end product of immune activation, has shown promise as a therapeutic target. IL-1 receptor antagonists such as Anakinra have previously been employed as a treatment strategy in SOD1 mice, yielding beneficial effects in survival and motor functions (Meissner et al., 2010). This *in vivo* success has led to at least one preclinical trial to determine the safety profile of the drug and to investigate its effects on inflammatory biomarkers. After treatment with Anakinra for 1 year, individuals did not show a statistically significant improvement in disease progression compared to a historical control cohort. Inflammatory markers including IL-6 and TNF-α both decreased at the 6 month time point; however statistical significance was not achieved, to which the authors attribute to the small sample size. Interestingly, participants in the trial generated antibodies against Anakinra after the 6 month time point, potentially preventing efficacy (Maier et al., 2015).

Additional investigations have been conducted on targeting NF-κB through the use of purified and pH adjusted sodium chlorite, NP001. In phase 1 testing of NP001, participants were monitored to determine the safety profile of the drug, as well as changes in markers of monocytic activation, CD16 and HLA-DR. Promisingly, NP001 was well-tolerated by participants at all tested doses and additionally lead to a decrease in HLA-DR, independent of dose, and a dose-dependent decrease in CD16 (Miller et al., 2014). When taken to phase 2 testing, the efficacy of NP001 was shown to be mixed among different subsets participants. Disease progression over the 6 month testing period was not significantly slowed across all individuals receiving NP001, however when classified by baseline inflammation, individual with a higher systemic level of inflammation before administration of NP001 exhibited a 41% reduction in disease progression (Miller et al., 2015).

Other routes of inhibiting inflammation in ALS have recently also been investigated. S100A4, a Ca^2+^ binding protein and DAMP, which has previously been demonstrated to play an essential role in multiple cellular processes, has been targeted as a treatment target. Similar to the temporal pattern of activation of microglia, in mutant SOD1 rats, S100A4 exhibits a significant increase in concentration in the pre-symptomatic phase ALS, and remains elevated throughout the disease course. In recent work in primary microglia derived from SOD1 mice, niclosamide, a transcriptional inhibitor of S100A4, was shown to have beneficial effects in the inhibition of NOX2, among other pro-inflammatory mechanisms in microglia (Serrano et al., 2019).

## Conclusion

The intronic HRE of C9orf72 has a vast number of pathologies that ultimately lead to the development of ALS. A major, underexplored facet of the pathogenesis of the disease is the activation of the immune system by the various biochemical and molecular immediate effects of the expansion. This untapped direction may serve as a potential target for novel therapeutics targeting the immune effects of the expansion to either delay or ultimately prevent neurodegeneration. One major obstacle to exploring this route of treatment is early detection of ALS, increasing the mounting need for novel biomarkers of early disease progression. With inflammation being present in other forms of ALS, these therapeutic targets may additionally be extrapolated to other forms of the disease to improve outlook for individuals afflicted with the disorder.

## Author Contributions

KT wrote the manuscript. KT, CS, FH, KO, and GP conceptualized and edited the manuscript and approved this work for publication.

## Conflict of Interest

The authors declare that the research was conducted in the absence of any commercial or financial relationships that could be construed as a potential conflict of interest.

## Abbreviations

4-HNE: 4-hydroxy-2-non-enal
8-oxo-dG: 8-oxo-2 ′ -desoxyguanosine
ALS: amyotrophic lateral sclerosis
ASC: apoptosis-associated speck like protein containing a caspase recruitment domain
c9ALS/FTD: C9orf72 amyotrophic lateral sclerosis/frontotemporal dementia
C9orf72: chromosome 9 open reading frame 72
COX: cyclooxygenase
DPR: dipeptide repeat protein
fALS: familial ALS
FTD: frontotemporal dementia
GFAP: glial fibrillary acidic protein
HNRNPA1: heterogeneous nuclear ribonucleoprotein A1
HRE: hexanucleotide repeat expansion
IgG: immunoglobulin G
LPS: lipopolysaccharide
NfL: neurofilament light chain
NMJ: neuromuscular junction
PBMC: peripheral blood mononuclear cell
poly-GA: poly(Glycine-Alanine)
poly-GP: Poly(Glycine-Proline)
poly-GR: poly(Glycine-Arginine)
poly-PA: poly(Proline-Alanine)
poly-PR: poly(Proline-Arginine)
RAN translation: repeat associated non-ATG dependent translation
RBP: ribosomal binding protein
RNP: ribonucleoprotein
RNS: reactive nitrogen species
ROS: reactive oxygen species
SGs: stress granules
SOD1: superoxide dismutase 1
TBPH: TAR DNA-binding protein-43 homolog
TDP-43: TAR DNA binding protein of 43 kDa.

## References

[B1] Allen S. P. Hall B. Woof R. Francis L. Gatto N. (2019). *C9orf72* expansion within astrocytes reduces metabolic flexibility in amyotrophic lateral sclerosis. *Brain* 1–20. 10.1093/brain/awz302 31647549 PMC6906594

[B2] Aubin N. Curet O. Deffois A. Carter C. (2002). Aspirin and salicylate protect against MPTP-induced dopamine depletion in mice. *J. Neurochem.* 71 1635–1642. 10.1046/j.1471-4159.1998.71041635.x 9751197

[B3] Balendra R. Isaacs A. M. (2018). C9orf72-mediated ALS and FTD: multiple pathways to disease. *Nat. Rev. Neurol.* 14 544–558. 10.1038/s41582-018-0047-2 30120348 PMC6417666

[B4] Barbarawi M. Kheiri B. Zayed Y. Gakhal I. Al-Abdouh A. Barbarawi O. (2019). Aspirin efficacy in primary prevention: a meta-analysis of randomized controlled trials. *High Blood Pressure Cardiovasc. Prevent.* 26 283–291. 10.1007/s40292-019-00325-5 31280451

[B5] Barnéoud P. Curet O. (1999). Beneficial effects of lysine acetylsalicylate, a soluble salt of aspirin, on motor performance in a transgenic model of amyotrophic lateral sclerosis. *Exp. Neurol.* 155 243–251. 10.1006/EXNR.1998.6984 10072299

[B6] Beers D. R. Appel S. H. (2019). Immune dysregulation in amyotrophic lateral sclerosis: mechanisms and emerging therapies. *Lancet Neurol.* 18 211–220. 10.1016/S1474-4422(18)30394-6 30663610

[B7] Beers D. R. Henkel J. S. Xiao Q. Zhao W. Wang J. Yen A. A. (2006). Wild-type microglia extend survival in PU.1 knockout mice with familial amyotrophic lateral sclerosis. *Proc. Natl. Acad. Sci. U.S.A.* 103 16021–16026. 10.1073/pnas.0607423103 17043238 PMC1613228

[B8] Bélanger M. Allaman I. Magistretti P. J. (2011). Brain energy metabolism: focus on astrocyte-neuron metabolic cooperation. *Cell Metab.* 14 724–738. 10.1016/j.cmet.2011.08.016 22152301

[B9] Betteridge D. J. (2000). What is oxidative stress? *Metab. Clin. Exp.* 49 3–8. 10.1016/S0026-0495(00)80077-3 10693912

[B10] Bochman M. L. Paeschke K. Zakian V. A. (2012). DNA secondary structures: stability and function of G-quadruplex structures. *Nat. Rev. Genet.* 13 770–780. 10.1038/nrg3296 23032257 PMC3725559

[B11] Brettschneider J. Toledo J. B. Van Deerlin V. M. Elman L. McCluskey L. Lee V. M. Y. (2012). Microglial activation correlates with disease progression and upper motor neuron clinical symptoms in amyotrophic lateral sclerosis. *PLoS One* 7:e39216. 10.1371/journal.pone.0039216 22720079 PMC3375234

[B12] Butovsky O. Jedrychowski M. P. Cialic R. Krasemann S. Murugaiyan G. Fanek Z. (2015). Targeting miR-155 restores abnormal microglia and attenuates disease in SOD1 mice. *Ann. Neurol.* 77 75–99. 10.1002/ana.24304 25381879 PMC4432483

[B13] Byrne S. Heverin M. Elamin M. Walsh C. Hardiman O. (2014). Intermediate repeat expansion length in C9orf72 may be pathological in amyotrophic lateral sclerosis. *Amyotroph. Lateral Scler. Frontotemporal Degener.* 15 148–150. 10.3109/21678421.2013.838586 24053774

[B14] Campanari M. L. García-Ayllón M. S. Ciura S. Sáez-Valero J. Kabashi E. (2016). Neuromuscular junction impairment in amyotrophic lateral sclerosis: reassessing the role of acetylcholinesterase. *Front. Mol. Neurosci.* 9:160. 10.3389/FNMOL.2016.00160 28082868 PMC5187284

[B15] Chen L. Liu B. (2017). Relationships between stress granules, oxidative stress, and neurodegenerative diseases. *Oxid. Med. Cell. Long.* 2017:1809592. 10.1155/2017/1809592 28194255 PMC5286466

[B16] Cheng W. Wang S. Mestre A. A. Fu C. Makarem A. Xian F. (2018). C9ORF72 GGGGCC repeat-associated non-AUG translation is upregulated by stress through eIF2α phosphorylation. *Nat. Commun.* 9:51. 10.1038/s41467-017-02495-z 29302060 PMC5754368

[B17] Chew J. Cook C. Gendron T. F. Jansen-West K. Del Rosso G. Daughrity L. M. (2019). Aberrant deposition of stress granule-resident proteins linked to C9orf72-associated TDP-43 proteinopathy. *Mol. Neurodegen.* 14:9. 10.1186/s13024-019-0310-z 30767771 PMC6377782

[B18] Chitiprolu M. Jagow C. Tremblay V. Bondy-Chorney E. Paris G. Savard A. (2018). A complex of C9ORF72 and p62 uses arginine methylation to eliminate stress granules by autophagy. *Nat. Commun.* 9:2794. 10.1038/s41467-018-05273-7 30022074 PMC6052026

[B19] Cleary J. D. Pattamatta A. Ranum L. P. W. (2018). Repeat-associated non-ATG (RAN) translation. *J. Biol. Chem.* 293 16127–16141. 10.1074/jbc.R118.003237 30213863 PMC6200949

[B20] Colombrita C. Zennaro E. Fallini C. Weber M. Sommacal A. Buratti E. (2009). TDP-43 is recruited to stress granules in conditions of oxidative insult. *J. Neurochem.* 111 1051–1061. 10.1111/j.1471-4159.2009.06383.x 19765185

[B21] Corcia P. Tauber C. Vercoullie J. Arlicot N. Prunier C. Praline J. (2012). Molecular imaging of microglial activation in amyotrophic lateral sclerosis. *PLoS One* 7:e52941. 10.1371/JOURNAL.PONE.0052941 23300829 PMC3534121

[B22] Cruts M. Gijselinck I. Van Der Zee J. Engelborghs S. Wils H. Pirici D. (2006). Null mutations in progranulin cause ubiquitin-positive frontotemporal dementia linked to chromosome 17q21. *Nature* 442 920–924. 10.1038/nature05017 16862115

[B23] Cruz M. P. (2018). Edaravone (Radicava): a novel neuroprotective agent for the treatment of amyotrophic lateral sclerosis. *P T* 43 25–28. 29290672 PMC5737249

[B24] Cudkowicz M. E. Shefner J. M. Schoenfeld D. A. Zhang H. Andreasson K. I. Rothstein J. D. (2006). Trial of celecoxib in amyotrophic lateral sclerosis. *Ann. Neurol.* 60 22–31. 10.1002/ana.20903 16802291

[B25] Deora V. Lee J. D. Albornoz E. A. McAlary L. Jagaraj C. J. Robertson A. A. B. (2019). The microglial NLRP3 inflammasome is activated by amyotrophic lateral sclerosis proteins. *GLIA* [Epub ahead of print]. 10.1002/glia.23728 31596526

[B26] Devos D. Moreau C. Kyheng M. Garçon G. Rolland A. S. Blasco H. (2019). A ferroptosis–based panel of prognostic biomarkers for Amyotrophic Lateral Sclerosis. *Sci. Rep.* 9:2918. 10.1038/s41598-019-39739-5 30814647 PMC6393674

[B27] Drachman D. B. Frank K. Dykes-Hoberg M. Teismann P. Almer G. Przedborski S. (2002). Cyclooxygenase 2 inhibition protects motor neurons and prolongs survival in a transgenic mouse model of ALS. *Ann. Neurol.* 52 771–778. 10.1002/ana.10374 12447931

[B28] Dubbelaar M. L. Kracht L. Eggen B. Boddeke E. W. G. M. (2018). The kaleidoscope of microglial phenotypes. *Front. Immunol.* 9:17531. 10.3389/fimmu.2018.01753 30108586 PMC6079257

[B29] Fay M. M. Anderson P. J. Ivanov P. (2017). ALS/FTD-associated C9ORF72 repeat RNA promotes phase transitions in vitro and in cells. *Cell Rep.* 21 3573–3584. 10.1016/j.celrep.2017.11.093 29262335 PMC5741101

[B30] Ferrante R. J. Browne S. E. Shinobu L. A. Bowling A. C. Jay Baik M. Macgarvey U. (1997). Evidence of increased oxidative damage in both sporadic and familial amyotrophic lateral sclerosis. *J. Neurochem.* 69 2064–2074. 10.1046/j.1471-4159.1997.69052064.x 9349552

[B31] Fischer R. Maier O. (2015). Interrelation of oxidative stress and inflammation in neurodegenerative disease: role of TNF. *Oxid. Med. Cell. Long.* 2015 1–18. 10.1155/2015/610813 25834699 PMC4365363

[B32] Fitzpatrick F. (2004). Cyclooxygenase enzymes: regulation and function. *Curr. Pharmaceut. Design* 10 577–588. 10.2174/1381612043453144 14965321

[B33] Fratta P. Mizielinska S. Nicoll A. J. Zloh M. Fisher E. M. C. Parkinson G. (2012). C9orf72 hexanucleotide repeat associated with amyotrophic lateral sclerosis and frontotemporal dementia forms RNA G-quadruplexes. *Sci. Rep.* 2:1016. 10.1038/srep01016 23264878 PMC3527825

[B34] Freibaum B. D. Taylor J. P. (2017). The role of dipeptide repeats in C9ORF72-Related ALS-FTD. *Front. Mol. Neurosci.* 10:35. 10.3389/fnmol.2017.00035 28243191 PMC5303742

[B35] Gao H.-M. Zhou H. Hong J. S. (2012). NADPH oxidases: novel therapeutic targets for neurodegenerative diseases. *Trends Pharmacol. Sci.* 33 295–303. 10.1016/j.tips.2012.03.008 22503440 PMC3477578

[B36] Garbuzova-Davis S. Sanberg P. R. (2014). Blood-CNS barrier impairment in ALS patients versus an animal model. *Front. Cell. Neurosci.* 8:21. 10.3389/fncel.2014.00021 24550780 PMC3910123

[B37] Garwood C. J. Ratcliffe L. E. Simpson J. E. Heath P. R. Ince P. G. Wharton S. B. (2017). Review: astrocytes in Alzheimer’s disease and other age-associated dementias: a supporting player with a central role. *Neuropathol. Appl. Neurobiol.* 43 281–298. 10.1111/nan.12338 27442752

[B38] Geloso M. C. Corvino V. Marchese E. Serrano A. Michetti F. D’Ambrosi N. (2017). The dual role of microglia in ALS: mechanisms and therapeutic approaches. *Front. Aging Neurosci.* 9:242. 10.3389/fnagi.2017.00242 28790913 PMC5524666

[B39] Gijselinck I. Van Mossevelde S. Van Der Zee J. Sieben A. Engelborghs S. De Bleecker J. (2016). The C9orf72 repeat size correlates with onset age of disease, DNA methylation and transcriptional downregulation of the promoter. *Mol. Psychiatry* 21 1112–1124. 10.1038/mp.2015.159 26481318 PMC4960451

[B40] Haeusler A. R. Donnelly C. J. Periz G. Simko E. A. J. Shaw P. G. Kim M. S. (2014). C9orf72 nucleotide repeat structures initiate molecular cascades of disease. *Nature* 507 195–200. 10.1038/nature13124 24598541 PMC4046618

[B41] Herman F. J. Pasinetti G. M. (2018). Principles of inflammasome priming and inhibition: implications for psychiatric disorders. *Brain Behav. Immun.* 73 66–84. 10.1016/j.bbi.2018.06.010 29902514 PMC6526722

[B42] Herrmann F. Fackelmayer F. O. (2009). Nucleo-cytoplasmic shuttling of protein arginine methyltransferase 1 (PRMT1) requires enzymatic activity. *Genes Cells* 14 309–317. 10.1111/j.1365-2443.2008.01266.x 19170758

[B43] Italiani P. Carlesi C. Giungato P. Puxeddu I. Borroni B. Bossù P. (2014). Evaluating the levels of interleukin-1 family cytokines in sporadic amyotrophic lateral sclerosis. *J. Neuroinflamm.* 11:94. 10.1186/1742-2094-11-94 24884937 PMC4039322

[B44] Iyer S. Subramanian V. Acharya K. R. (2018). C9orf72, a protein associated with amyotrophic lateral sclerosis (ALS) is a guanine nucleotide exchange factor. *PeerJ* 6:e5815. 10.7717/peerj.5815 30356970 PMC6195791

[B45] Johann S. Heitzer M. Kanagaratnam M. Goswami A. Rizo T. Weis J. (2015). NLRP3 inflammasome is expressed by astrocytes in the SOD1 mouse model of ALS and in human sporadic ALS patients. *Glia* 63 2260–2273. 10.1002/glia.22891 26200799

[B46] Kedersha N. Anderson P. (2007). Mammalian stress granules and processing bodies. *Methods Enzymol.* 431 61–81. 10.1016/S0076-6879(07)31005-7 17923231

[B47] Keller A. F. Gravel M. Kriz J. (2011). Treatment with minocycline after disease onset alters astrocyte reactivity and increases microgliosis in SOD1 mutant mice. *Exp. Neurol.* 228 69–79. 10.1016/j.expneurol.2010.12.010 21168408

[B48] Kim G. H. Kim J. E. Rhie S. J. Yoon S. (2015). The role of oxidative stress in neurodegenerative diseases. *Exp. Neurobiol.* 24 325–340. 10.5607/en.2015.24.4.325 26713080 PMC4688332

[B49] Kim H.-J. Raphael A. R. LaDow E. S. McGurk L. Weber R. A. Trojanowski J. Q. (2014). Therapeutic modulation of eIF2α phosphorylation rescues TDP-43 toxicity in amyotrophic lateral sclerosis disease models. *Nat. Genet.* 46 152–160. 10.1038/ng.2853 24336168 PMC3934366

[B50] Koppers M. Blokhuis A. M. Westeneng H.-J. Terpstra M. L. Zundel C. A. C. Vieira de Sá R. (2015). C9orf72 ablation in mice does not cause motor neuron degeneration or motor deficits. *Ann. Neurol.* 78 426–438. 10.1002/ana.24453 26044557 PMC4744979

[B51] Kreutzberg G. W. (1996). Microglia: a sensor for pathological events in the CNS. *Trends Neurosci.* 19 312–318. 10.1016/0166-2236(96)10049-7 8843599

[B52] Kriz J. Nguyen M. D. Julien J.-P. (2002). Minocycline slows disease progression in a mouse model of amyotrophic lateral sclerosis. *Neurobiol. Dis.* 10 268–278. 10.1006/NBDI.2002.0487 12270689

[B53] Kumar V. Kashav T. Islam A. Ahmad F. Hassan M. I. (2016). Structural insight into C9orf72 hexanucleotide repeat expansions: towards new therapeutic targets in FTD-ALS. *Neurochem. Int.* 100 11–20. 10.1016/J.NEUINT.2016.08.008 27539655

[B54] Kwon I. Xiang S. Kato M. Wu L. Theodoropoulos P. Wang T. (2014). Poly-dipeptides encoded by the C9orf72 repeats bind nucleoli, impede RNA biogenesis, and kill cells. *Science* 345 1139–1145. 10.1126/SCIENCE.1254917 25081482 PMC4459787

[B55] Lall D. Baloh R. H. (2017). Microglia and C9orf72 in neuroinflammation and ALS and frontotemporal dementia. *J. Clin. Invest.* 127 3250–3258. 10.1172/JCI90607 28737506 PMC5669558

[B56] Lee K.-H. Zhang P. Kim H. J. Mitrea D. M. Sarkar M. Freibaum B. D. (2016). C9orf72 dipeptide repeats impair the assembly, dynamics, and function of membrane-less organelles. *Cell* 167 774.e17–788.e17. 10.1016/j.cell.2016.10.002 27768896 PMC5079111

[B57] Lee S. Huang E. J. (2017). Modeling ALS and FTD with iPSC-derived neurons. *Brain Res.* 1656 88–97. 10.1016/j.brainres.2015.10.003 26462653 PMC4833714

[B58] Li Q. Barres B. A. (2018). Microglia and macrophages in brain homeostasis and disease. *Nat. Rev. Immunol.* 18 225–242. 10.1038/nri.2017.125 29151590

[B59] Liao B. Zhao W. Beers D. R. Henkel J. S. Appel S. H. (2012). Transformation from a neuroprotective to a neurotoxic microglial phenotype in a mouse model of ALS. *Exp. Neurol.* 237 147–152. 10.1016/j.expneurol.2012.06.011 22735487 PMC4126417

[B60] Liu C. Geng Y. Miao H. Shi X. You Y. Xu N. (2019). G-quadruplex structures formed by human telomeric DNA and C9orf72 hexanucleotide repeats. *Biophys. Rev.* 11 389–393. 10.1007/s12551-019-00545-y 31127470 PMC6557930

[B61] Lomen-Hoerth C. Anderson T. Miller B. (2002). The overlap of amyotrophic lateral sclerosis and frontotemporal dementia. *Neurology* 59 1077–1079. 10.1212/WNL.59.7.1077 12370467

[B62] Lu C.-H. Allen K. Oei F. Leoni E. Kuhle J. Tree T. (2016). Systemic inflammatory response and neuromuscular involvement in amyotrophic lateral sclerosis. *Neuroimmunol. Neuroinflamm.* 3:e244. 10.1212/NXI.0000000000000244 27308305 PMC4897985

[B63] Luo L. Song Z. Li X. Huiwang, Zeng Y. Qinwang (2019). Efficacy and safety of edaravone in treatment of amyotrophic lateral sclerosis—a systematic review and meta-analysis. *Neurol. Sci.* 40 235–241. 10.1007/s10072-018-3653-2 30483992

[B64] Ma M. W. Wang J. Zhang Q. Wang R. Dhandapani K. M. Vadlamudi R. K. (2017). NADPH oxidase in brain injury and neurodegenerative disorders. *Mol. Neurodegen.* 12:7. 10.1186/s13024-017-0150-7 28095923 PMC5240251

[B65] Mackenzie I. R. Arzberger T. Kremmer E. Troost D. Lorenzl S. Mori K. (2013). Dipeptide repeat protein pathology in C9ORF72 mutation cases: clinico-pathological correlations. *Acta Neuropathol.* 126 859–879. 10.1007/s00401-013-1181-y 24096617

[B66] Maharjan N. Künzli C. Buthey K. Saxena S. (2017). C9ORF72 regulates stress granule formation and its deficiency impairs stress granule assembly, hypersensitizing cells to stress. *Mol. Neurobiol.* 54 3062–3077. 10.1007/s12035-016-9850-1 27037575

[B67] Mahoney C. J. Beck J. Rohrer J. D. Lashley T. Mok K. Shakespeare T. (2012). Frontotemporal dementia with the *C9ORF72* hexanucleotide repeat expansion: clinical, neuroanatomical and neuropathological features. *Brain* 135 736–750. 10.1093/brain/awr361 22366791 PMC3286330

[B68] Maier A. Deigendesch N. Müller K. Weishaupt J. H. Krannich A. Röhle R. (2015). Interleukin-1 antagonist Anakinra in amyotrophic lateral sclerosis - A pilot study. *PLoS One* 10:e0139684. 10.1371/journal.pone.0139684 26444282 PMC4596620

[B69] Mammana S. Fagone P. Cavalli E. Basile M. S. Petralia M. C. Nicoletti F. (2018). The role of macrophages in neuroinflammatory and neurodegenerative pathways of alzheimer’s disease, amyotrophic lateral sclerosis, and multiple sclerosis: pathogenetic cellular effectors and potential therapeutic targets. *Int. J. Mol. Sci.* 19:E831. 10.3390/ijms19030831 29533975 PMC5877692

[B70] McCauley M. E. Baloh R. H. (2019). Inflammation in ALS/FTD pathogenesis. *Acta Neuropathol.* 137 715–730. 10.1007/s00401-018-1933-9 30465257 PMC6482122

[B71] McCombe P. A. Henderson R. D. (2011). The role of immune and inflammatory mechanisms in ALS. *Curr. Mol. Med.* 11 246–254. 10.2174/156652411795243450 21375489 PMC3182412

[B72] Meissner F. Molawi K. Zychlinsky A. (2010). Mutant superoxide dismutase 1-induced IL-1beta accelerates ALS pathogenesis. *Proc. Natl. Acad. Sci. U.S.A.* 107 13046–13050. 10.1073/pnas.1002396107 20616033 PMC2919927

[B73] Miller R. G. Block G. Katz J. S. Barohn R. J. Gopalakrishnan V. Cudkowicz M. (2015). Randomized phase 2 trial of NP001-a novel immune regulator: safety and early efficacy in ALS. *Neurol. Neuroimmunol. NeuroInflamm.* 2:e100. 10.1212/NXI.0000000000000100 25884010 PMC4396529

[B74] Miller R. G. Zhang R. Block G. Katz J. Barohn R. Kasarskis E. (2014). NP001 regulation of macrophage activation markers in ALS: a phase I clinical and biomarker study. *Amyotroph. Lateral Scler. Frontotemporal Degener.* 15 601–609. 10.3109/21678421.2014.951940 25192333 PMC5524125

[B75] Mitchell D. J. O’brien M. R. Joshi M. Mitchell J. D. (2009). Audit of outcomes in motor neuron disease (MND) patients treated with riluzole Audit of outcomes in motor neuron disease (MND) patients treated with riluzole. *Amyotroph. Lateral Scler.* 7 67–71. 10.1080/14660820500396984 16753969

[B76] Mitchell J. C. Constable R. So E. Vance C. Scotter E. Glover L. (2015). Wild type human TDP-43 potentiates ALS-linked mutant TDP-43 driven progressive motor and cortical neuron degeneration with pathological features of ALS. *Acta Neuropathol. Commun.* 3 36. 10.1186/s40478-015-0212-4 26108367 PMC4479086

[B77] Mizielinska S. Grönke S. Niccoli T. Ridler C. E. Clayton E. L. Devoy A. (2014). C9orf72 repeat expansions cause neurodegeneration in *Drosophila* through arginine-rich proteins. *Science* 345 1192–1194. 10.1126/science.1256800 25103406 PMC4944841

[B78] Moens T. G. Niccoli T. Wilson K. M. Atilano M. L. Birsa N. Gittings L. M. (2019). C9orf72 arginine-rich dipeptide proteins interact with ribosomal proteins in vivo to induce a toxic translational arrest that is rescued by eIF1A. *Acta Neuropathol.* 137 487–500. 10.1007/s00401-018-1946-4 30604225 PMC6514073

[B79] Mori K. Weng S.-M. Arzberger T. May S. Rentzsch K. Kremmer E. (2013). The C9orf72 GGGGCC repeat is translated into aggregating dipeptide-repeat proteins in FTLD/ALS. *Science* 339 1335–1338. 10.1126/science.1232927 23393093

[B80] Müller N. (2019). COX-2 inhibitors, aspirin, and other potential anti-inflammatory treatments for psychiatric disorders. *Front. Psychiatry* 10:375. 10.3389/fpsyt.2019.00375 31214060 PMC6555131

[B81] Murray M. E. Dejesus-Hernandez M. Rutherford N. J. Baker M. Duara R. Graff-Radford N. R. (2011). Clinical and neuropathologic heterogeneity of c9FTD/ALS associated with hexanucleotide repeat expansion in *C9ORF72*. *Acta Neuropathol.* 122, 673–690. 10.1007/s00401-011-0907-y 22083254 PMC3277860

[B82] O’Rourke J. G. Bogdanik L. Yáñez A. Lall D. Wolf A. J. Muhammad A. K. M. G. (2016). C9orf72 is required for proper macrophage and microglial function in mice. *Science* 351 1324–1329. 10.1126/science.aaf1064 26989253 PMC5120541

[B83] Pasinelli P. Brown R. H. (2006). Molecular biology of amyotrophic lateral sclerosis: insights from genetics. *Nat. Rev. Neurosci.* 7 710–723. 10.1038/nrn1971 16924260

[B84] Pollari E. Goldsteins G. Bart G. Koistinaho J. Giniatullin R. (2014). The role of oxidative stress in degeneration of the neuromuscular junction in amyotrophic lateral sclerosis. *Front. Cell. Neurosci.* 8:131. 10.3389/fncel.2014.00131 24860432 PMC4026683

[B85] Pompl P. N. Ho L. Bianchi M. Mcmanus T. Qin W. PasinettI G. M. (2003). A therapeutic role for cyclooxygenase-2 inhibitors in a transgenic mouse model of amyotrophic lateral sclerosis. *FASEB J.* 17 725–727. 10.1096/fj.02-0876fje 12586733

[B86] Prado L. D. G. R. Bicalho I. C. S. Magalhães D. Caramelli P. Teixeira A. L. de Souza L. C. (2015). C9ORF72 and the FTD-ALS spectrum: a systematic review of neuroimaging studies. *Dementia Neuropsychol.* 9 413–421. 10.1590/1980-57642015DN94000413 29213991 PMC5619324

[B87] Prasad A. Bharathi V. Sivalingam V. Girdhar A. Patel B. K. (2019). Molecular mechanisms of TDP-43 misfolding and pathology in amyotrophic lateral sclerosis. *Front. Mol. Neurosci.* 12:25. 10.3389/fnmol.2019.00025 30837838 PMC6382748

[B88] Renton A. E. Chiò A. Traynor B. J. (2014). State of play in amyotrophic lateral sclerosis genetics. *Nat. Neurosci.* 17 17–23. 10.1038/nn.3584 24369373 PMC4544832

[B89] Rostalski H. Leskelä S. Huber N. Katisko K. Cajanus A. Solje E. (2019). Astrocytes and microglia as potential contributors to the pathogenesis of C9orf72 repeat expansion-associated FTLD and ALS. *Front. Neurosci.* 13:486. 10.3389/fnins.2019.00486 31156371 PMC6529740

[B90] Sellier C. Campanari M. Julie Corbier C. Gaucherot A. Kolb-Cheynel I. Oulad-Abdelghani M. (2016). Loss of C9ORF72 impairs autophagy and synergizes with polyQ Ataxin-2 to induce motor neuron dysfunction and cell death. *EMBO J.* 35 1276–1297. 10.15252/embj.201593350 27103069 PMC4910533

[B91] Serrano A. Apolloni S. Rossi S. Lattante S. Sabatelli M. Peric M. (2019). The S100A4 transcriptional inhibitor niclosamide reduces pro-inflammatory and migratory phenotypes of microglia: implications for amyotrophic lateral sclerosis. *Cells* 8:E1261. 10.3390/cells8101261 31623154 PMC6829868

[B92] Shemer A. Erny D. Jung S. Prinz M. (2015). Microglia plasticity during health and disease: an immunological perspective. *Trends Immunol.* 36 614–624. 10.1016/j.it.2015.08.003 26431939

[B93] Shin J. H. Lee Y. A. Lee J. K. Lee Y. B. Cho W. Im D. S. (2012). Concurrent blockade of free radical and microsomal prostaglandin E synthase-1-mediated PGE2 production improves safety and efficacy in a mouse model of amyotrophic lateral sclerosis. *J. Neurochem.* 122 952–961. 10.1111/j.1471-4159.2012.07771.x 22537108

[B94] Solomon D. A. Stepto A. Au W. H. Adachi Y. Diaper D. C. Hall R. (2018). A feedback loop between dipeptide-repeat protein, TDP-43 and karyopherin-α mediates C9orf72-related neurodegeneration. *Brain* 141 2908–2924. 10.1093/brain/awy241 30239641 PMC6158706

[B95] Strong M. J. Lomen-Hoerth C. Caselli R. J. Bigio E. H. Yang W. (2003). Cognitive impairment, frontotemporal dementia, and the motor neuron diseases. *Ann. Neurol.* 54 S20–S23. 10.1002/ana.10574 12833364

[B96] Swanson K. V. Deng M. Ting J. P.-Y. (2019). The NLRP3 inflammasome: molecular activation and regulation to therapeutics. *Nat. Rev. Immunol.* 19 477–489. 10.1038/s41577-019-0165-0 31036962 PMC7807242

[B97] Takada L. T. (2015). The genetics of monogenic frontotemporal dementia. *Dementia Neuropsychol.* 9 219–229. 10.1590/1980-57642015DN93000003 29213965 PMC5619362

[B98] Thys R. G. Wang Y.-H. (2015). DNA replication dynamics of the GGGGCC repeat of the C9orf72 gene. *J. Biol. Chem.* 290 28953–28962. 10.1074/jbc.M115.660324 26463209 PMC4661408

[B99] Tsai C.-P. Lin F.-C. Lee J. K.-W. Lee C. T.-C. (2015). Aspirin use associated with amyotrophic lateral sclerosis: a total population-based case-control study. *J. Epidemiol.* 25 172–177. 10.2188/jea.JE20140070 25721071 PMC4310879

[B100] van Blitterswijk M. Gendron T. F. Baker M. C. DeJesus-Hernandez M. Finch N. A. Brown P. H. (2015). Novel clinical associations with specific C9ORF72 transcripts in patients with repeat expansions in C9ORF72. *Acta Neuropathol.* 130 863–876. 10.1007/s00401-015-1480-6 26437865 PMC4655160

[B101] Vane J. R. Botting R. M. (2003). The mechanism of action of aspirin. *Thromb. Res.* 110 255–258. 10.1016/s0049-3848(03)00379-7 14592543

[B102] Varcianna A. Myszczynska M. A. Castelli L. M. O’Neill B. Kim Y. Talbot J. (2019). Micro-RNAs secreted through astrocyte-derived extracellular vesicles cause neuronal network degeneration in C9orf72 ALS. *EBioMedicine* 40 626–635. 10.1016/j.ebiom.2018.11.067 30711519 PMC6413467

[B103] Volonté C. Amadio S. Fabbrizio P. Apolloni S. (2019). Functional microglia neurotransmitters in amyotrophic lateral sclerosis. *Semin. Cell Dev. Biol.* 94 121–128. 10.1016/j.semcdb.2019.04.014 31009755

[B104] Westergard T. McAvoy K. Russell K. Wen X. Pang Y. Morris B. (2019). Repeat-associated non-AUG translation in C9orf72-ALS/FTD is driven by neuronal excitation and stress. *EMBO Mol. Med.* 11:e9423. 10.15252/emmm.201809423 30617154 PMC6365928

[B105] Winter A. N. Brenner M. C. Punessen N. Snodgrass M. Byars C. Arora Y. (2017). Comparison of the neuroprotective and anti-inflammatory effects of the anthocyanin metabolites, protocatechuic acid and 4-hydroxybenzoic acid. *Oxid. Med. Cell. Long.* 2017:6297080. 10.1155/2017/6297080 28740571 PMC5504963

[B106] Winton M. J. Igaz L. M. Wong M. M. Kwong L. K. Trojanowski J. Q. Lee V. M.-Y. (2008). Disturbance of nuclear and cytoplasmic TAR DNA-binding protein (TDP-43) induces disease-like redistribution, sequestration, and aggregate formation. *J. Biol. Chem.* 283 13302–13309. 10.1074/jbc.M800342200 18305110 PMC2442318

[B107] Xu W. Xu J. (2018). C9orf72 dipeptide repeats cause selective neurodegeneration and cell-autonomous excitotoxicity in *Drosophila* glutamatergic neurons. *J. Neurosci.* 38 7741–7752. 10.1523/JNEUROSCI.0908-18.2018 30037833 PMC6705968

[B108] Yoshino H. Kimura A. (2006). Investigation of the therapeutic effects of edaravone, a free radical scavenger, on amyotrophic lateral sclerosis (Phase II study). *Amyotroph. Lateral Scler.* 7 247–251. 10.1080/17482960600881870 17127563

[B109] Yuan Y. Zha H. Rangarajan P. Ling E.-A. Wu C. (2014). Anti-inflammatory effects of Edaravone and Scutellarin in activated microglia in experimentally induced ischemia injury in rats and in BV-2 microglia. *BMC Neurosci.* 15:125. 10.1186/s12868-014-0125-3 25416145 PMC4247200

[B110] Zhang P. Li W. Li L. Wang N. Li X. Gao M. (2012). Treatment with edaravone attenuates ischemic brain injury and inhibits neurogenesis in the subventricular zone of adult rats after focal cerebral ischemia and reperfusion injury. *Neuroscience* 201 297–306. 10.1016/j.neuroscience.2011.11.005 22116052

[B111] Zhang Y. Chen K. Sloan S. A. Bennett M. L. Scholze A. R. O’Keeffe S. (2014). An RNA-sequencing transcriptome and splicing database of glia, neurons, and vascular cells of the cerebral cortex. *J. Neurosci.* 34 11929–11947. 10.1523/JNEUROSCI.1860-14.2014 25186741 PMC4152602

[B112] Zhang Y. J. Jansen-West K. Xu Y.-F. Gendron T. F. Bieniek K. F. Lin W.-L. (2014). Aggregation-prone c9FTD/ALS poly(GA) RAN-translated proteins cause neurotoxicity by inducing ER stress. *Acta Neuropathol.* 128 505–524. 10.1007/s00401-014-1336-5 25173361 PMC4159567

[B113] Zhang Y. J. Gendron T. F. Ebbert M. T. W. O’Raw A. D. Yue M. Jansen-West K. (2018). Poly(GR) impairs protein translation and stress granule dynamics in C9orf72-associated frontotemporal dementia and amyotrophic lateral sclerosis. *Nat. Med.* 24 1136–1142. 10.1038/s41591-018-0071-1 29942091 PMC6520050

[B114] Zhang Y.-J. Guo L. Gonzales P. K. Gendron T. F. Wu Y. Jansen-West K. (2019). Heterochromatin anomalies and double-stranded RNA accumulation underlie C9orf 72 poly(PR) toxicity. *Science* 363:eaav2606. 10.1126/science.aav2606 30765536 PMC6524780

[B115] Zhao M. Kim J. R. van Bruggen R. Park J. (2018). RNA-binding proteins in amyotrophic lateral sclerosis. *Mol. Cells* 41 818–829. 10.14348/molcells.2018.0243 30157547 PMC6182225

[B116] Zhao W. Beers D. R. Bell S. Wang J. Wen S. Baloh R. H. (2015). TDP-43 activates microglia through NF-κB and NLRP3 inflammasome. *Exp. Neurol.* 273 24–35. 10.1016/j.expneurol.2015.07.019 26222336

[B117] Zhao W. Xie W. Beers D. R. Henkel J. S. Simpson E. P. Yen A. A. (2004). Activated microglia initiate motor neuron injury by a nitric oxide and glutamate-mediated mechanism. *J. Neuropathol. Exp. Neurol.* 63 964–977. 10.1093/jnen/63.9.964 15453095

[B118] Zhou B. Geng Y. Liu C. Miao H. Ren Y. Xu N. (2018). Characterizations of distinct parallel and antiparallel G-quadruplexes formed by two-repeat ALS and FTD related GGGGCC sequence. *Sci. Rep.* 8:2366. 10.1038/s41598-018-20852-w 29402965 PMC5799222

